# Socioeconomic and racial/ethnic differentials of C-reactive protein levels: a systematic review of population-based studies

**DOI:** 10.1186/1471-2458-7-212

**Published:** 2007-08-17

**Authors:** Aydin Nazmi, Cesar G Victora

**Affiliations:** 1 Federal University of Pelotas, Department of Social Medicine, Post-Graduate Program in Epidemiology, Rua Marechal Deodoro 1160, Pelotas, RS, 96020, Brazil

## Abstract

**Background:**

Socioeconomic and racial/ethnic factors strongly influence cardiovascular disease outcomes and risk factors. C-reactive protein (CRP), a non-specific marker of inflammation, is associated with cardiovascular risk, and knowledge about its distribution in the population may help direct preventive efforts. A systematic review was undertaken to critically assess CRP levels according to socioeconomic and racial/ethnic factors.

**Methods:**

Medline was searched through December 2006 for population-based studies examining CRP levels among adults with respect to indicators of socioeconomic position (SEP) and/or race/ethnicity. Bibliographies from located studies were scanned and 26 experts in the field were contacted for unpublished work.

**Results:**

Thirty-two relevant articles were located. Cross-sectional (n = 20) and cohort studies (n = 11) were included, as was the control group of one trial. CRP levels were examined with respect to SEP and race/ethnicity in 25 and 15 analyses, respectively. Of 20 studies that were unadjusted or adjusted for demographic variables, 19 found inverse associations between CRP levels and SEP. Of 15 similar studies, 14 found differences between racial/ethnic groups such that whites had the lowest while blacks, Hispanics and South Asians had the highest CRP levels. Most studies also included adjustment for potential mediating variables in the causal chain between SEP or race/ethnicity and CRP. Most of these studies showed attenuated but still significant associations.

**Conclusion:**

Increasing poverty and non-white race was associated with elevated CRP levels among adults. Most analyses in the literature are underestimating the true effects of racial/ethnic and socioeconomic factors due to adjustment for mediating factors.

## Background

The global burden of cardiovascular disease (CVD) represents the highest cause of mortality and one of the highest causes of morbidity both in high-income and low/middle-income countries [1,2]. It is well known that socioeconomic factors and race/ethnicity influence CVD outcomes and risk factors. Studies have consistently found inverse and independent associations between socioeconomic position (SEP) and the prevalence and incidence of CVD [3-8]. Multi-ethnic studies have pointed to significant differences between racial/ethnic groups [3,4,7,9,10]. In a Canadian study, for example, South Asians were shown to have 6 and 9% higher prevalence of CVD when compared to Europeans and Chinese [9], whereas in the US, heart disease mortality accounted for up to three times more deaths in blacks as compared to Asians [4]. The mechanisms that drive these socio-demographic risk factors to influence CVD are not fully understood, as differences in body mass index, smoking and other traditional risk factors fail to totally account for these associations. Therefore other mediating factors likely play significant roles in the relationship between sociodemographic characteristics and CVD.

Atherosclerosis, the process leading to coronary heart disease (CHD), has been described as an inflammatory disease [11]. Over the past decade, low-grade inflammation has widely been investigated as a candidate linking the association between traditional risk factors and CHD. An acute-phase protein produced by hepatocytes, C-reactive protein (CRP) has historically been used as a non-specific marker for infection, inflammation or tissue damage [12]. More recently, highly sensitive assays have permitted evaluation of elevated, but not acute, CRP levels in the assessment of risk for several chronic diseases.

Observational studies show that CRP levels are associated with future risk of chronic diseases including CHD [13-17] and diabetes [18-20] in apparently healthy people. Furthermore, CRP has been shown to add to the predictive value of conventional markers such as cholesterol [21] and blood pressure [22] in defining risk for coronary events. These associations, though consistent, have been described to exist to varying degrees.

Some authors have implicated CRP as an independent determinant of the disease process by actively promoting the proinflammatory phenotype [23,24]. Others are skeptical of this causal association, while not discounting that CRP may be proinflammatory under certain circumstances [25]. Researchers utilizing Mendelian Randomization techniques have found that certain genotypes are associated with higher CRP levels but that individuals with these genotypes are not necessarily at increased risk for cardiovascular events [26-29]. This calls into question the assumption that CRP levels are, per se, causally associated with risk for CHD. Nevertheless, the role of CRP as a risk marker is clear.

In order to adequately investigate the role of inflammation with respect to the processes that lead to CVD and ultimately to design effective interventions in the prevention of CVD based on these principles, the determinants of elevated CRP levels should be identified. A systematic review of the literature was undertaken to critically assess the evidence between socioeconomic and racial/ethnic factors and CRP levels among adults.

## Methods

### Search strategy

Three strategies to locate suitable articles were employed. First, the Medline (PubMed) database was searched. The main keyword search employed was "(C-reactive protein OR CRP OR high-sensitivity C-reactive protein OR hs-CRP) AND (socioeconomic OR race OR ethnic* OR education OR income OR determinants)". Articles published through December 2006 were considered. No language restrictions were used. Second, reference lists in the studies identified were scanned and if relevant were sought and reviewed. Finally, 26 experts in the field were contacted for unpublished or in-progress work, in addition to being contacted for clarification of findings from published studies.

### Dependent and independent variables

The dependent variable was CRP level. Studies using either high-sensitivity or conventional CRP assays were considered. Studies reporting arithmetic means (or when the type of mean is not specified) are described in terms of "means" whereas studies reporting geometric means are described as such. Authors either log-transformed the continuous CRP variable for normality prior to analysis or analyzed it as a categorical variable; the methods of one study could not be verified (Anand, 2004).

The independent variables of interest were socioeconomic position (SEP) and race/ethnicity. The term socioeconomic position covers a wide range of measures including, but not limited to, education, income, possession of assets, and index-based measures that inventory a number of socioeconomic factors and create a relative score. Studies that incorporated any socioeconomic variables were considered, given that they met the other inclusion criteria. For simplicity, SEP shall be used throughout this review when making general references. Studies that analyzed any racial or ethnic variables such as skin color or ethnic background were considered.

### Inclusion criteria

The review was restricted to population-based studies, including those employing methods such as random or probability-based sampling based on a given geographical area. Studies based on selected sample populations, including occupational cohorts, clinic-based or "convenience samples", were not considered. Studies that sampled either the entire population or specific age groups were included, provided that individuals were aged at least 17 years. Authors were contacted if sufficient methodological or analytical information was not provided in the article text or for possible overlapping information from the same samples in different articles.

### Conceptual model

In epidemiological models, the causal, mediating and confounding factors related to risk for disease and disease status can be complex to interpret. Conceptual frameworks aid in the organization of associated proximal and distal factors, helping to define which variables may constitute confounders and which ones are likely mediators [30]. Most studies are unclear about conceptual models used in statistical analyses and interpretations of findings [30,31].

**Figure 1:**
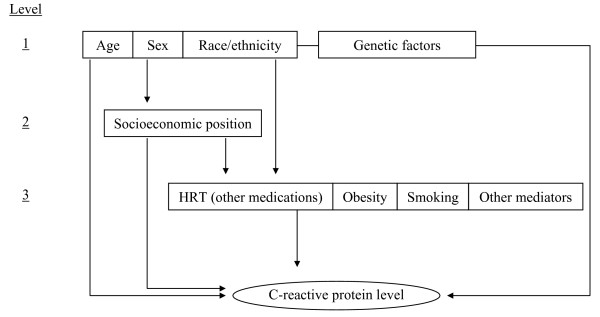
Conceptual model for the associations between race/ethnicity, socioeconomic position and C-reactive protein level.

We proposed a hierarchical model for the factors associated with CRP levels (Figure 1). In this model, variables on higher levels were considered as possible confounders for those on lower levels. The arrows represent possible pathways. For example, the association between SEP and CRP level may be confounded by the variables on level 1, since these are independently associated with both the dependent (SEP) and independent (CRP level) variables. On the other hand, level 3 variables may be on the causal pathway from SEP to CRP level and as such are possible mediators. In analyzing the association between SEP and CRP level, for instance, adjusting for smoking or obesity (treating them as confounders) would underestimate the true effect of SEP on CRP level by removing the effects mediated through smoking and obesity, which are on the causal pathway.

## Results

**Figure 2:**
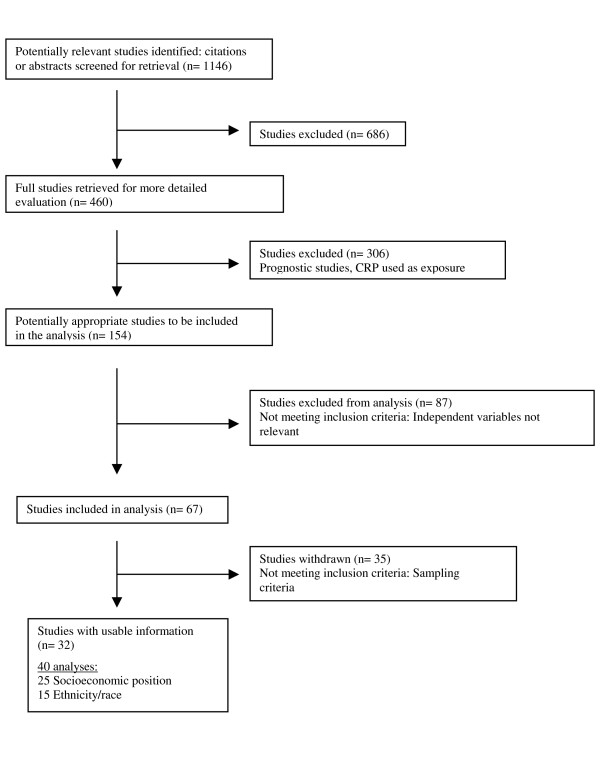
Number of studies associated with process of study selection (according to QUOROM guidelines).

Figure 2 shows a flowchart according to QUOROM statement guidelines outlining the number of articles identified at each step of the literature search [32]. Initial searching identified 1146 articles and 460 were retrieved for more detail from which 154 potentially appropriate articles were reviewed. Eighty-seven studies were excluded, primarily because CRP was analyzed as an independent variable in prognostic analyses, rather than as an outcome. Finally, 35 articles were withdrawn because they were not population-based, leaving 32 articles to be included in the review.

These studies were published between 1996 and 2006 and were conducted in the USA (14 studies; n = 96746), the UK (eight studies; n = 11049), Finland (two studies; n = 3793), Greece (two studies; n = 5313), Germany (two studies; n = 2891), Canada (n = 1250), Italy (n = 1650), Turkey (n = 1046) and New Zealand (n = 822). Of these, 25 analyses included SEP as an independent variable and 15 included ethnicity/race. Given that eight studies analyzed both independent variables, Additional file 1 includes 40 analyses from the 32 included studies. There were 20 cross-sectional and 11 cohort studies, all of which were analyzed as cross-sectional; two of the latter also included retrospective analyses of SEP in early life. One article reported on the control group of a larger trial study.

Different types of adjustment for covariates were used. In light of the conceptual model, analyses that were either unadjusted or adjusted for demographic confounding factors (including age, sex and race/ethnicity) are referred to as "minimally adjusted" models. Analyses that also included adjustment for potential mediating factors in addition to demographic confounders are referred to as "fully adjusted" models. In the results section of Additional file 1, the findings from the fully adjusted models appear on the bottom row, while those from minimally adjusted models appear on the top row. Some studies in which CRP level was not the main outcome showed only unadjusted distributions of CRP levels without statistical analysis. A formal quantitative analyses of this association (a meta-analysis) was not performed because given variability in exposure and outcome definitions such analyses were not possible. Additional file 2 presents effect sizes from the studies, providing numerical description of the data when available.

In the next sections we describe the main results of the studies reviewed. We comment on the statistical significance of the findings and provide the effect sizes and confidence intervals for the main analysis in each paper, when available in the publication.

### Socioeconomic status

A wide range of socioeconomic factors were tested, the most common being level of formal education and social class, based on composite scores or proxy measures. A total of 77467 analyses were represented. The Greek and Scottish studies examined the same individuals more than once; the former used different indicators of SEP in each assessment. Some studies using representative national samples (National Health and Nutrition Examination Survey, NHANES) from the USA also examined data from the same group of individuals more than once.

Of the 25 studies reporting on any SEP measure, nine presented only minimally adjusted results [20,26,33-39]; 12 also presented fully adjusted models [40-51]; and four only presented the latter [52-55]. Of the 21 studies presenting minimally adjusted results – 14 unadjusted and seven adjusted for demographic confounders – all but one showed inverse associations between SEP and CRP [20,26,33-37,39-51]. All 12 studies presenting both minimally and fully adjusted analyses found inverse associations in the former; in five of these the magnitude of the association decreased and was no longer statistically significant in the fully adjusted model [41,42,45,47,50]. The issue of whether the fully adjusted models may have included mediating factors is addressed in the Discussion.

Of the 16 [40-55] studies presenting multivariable analyses, nine found significant associations after adjusting for demographic, anthropometric and other postulated confounding variables [40,43,44,46,48,49,51,52,54].

One study using an unadjusted model [38] and seven using fully adjusted models [41,42,45,47,50,53,55] failed to find associations between any socioeconomic indicator and CRP. Direct associations were not found in any of the studies.

Education and/or index measures were examined in nearly all studies. We present these findings below as sub-groups of SEP analyses.

### Education

Fourteen studies examined the effect of educational indicators [20,33,35-37,40,42,43,45-47,49,52,53]. Of the 11 presenting minimally adjusted results [20,33,35-37,42,43,45-47,49] – six unadjusted and five adjusted for demographic variables – 10 found inverse associations, higher education being associated with lower CRP levels [20,33,35,37,42,43,45-47,49]. Three studies presenting only minimally adjusted results using NHANES data from 1988–1994 included potentially overlapping samples [33,36,37], but are discussed below as separate studies.

Among the nine studies that investigated this association in multivariable analysis [40,42,43,45-47,49,52,53], four found significant inverse associations with CRP levels after adjustment for postulated confounders such as age, smoking and body mass index (BMI = kg/m^2^) [40,46,49,52]. Three studies presenting fully adjusted results using NHANES data from 1999–2002 included potentially overlapping samples [40,52,53], but are discussed below as separate studies.

Of the four studies showing significant inverse associations in the fully adjusted analyses, three presented detailed information on effect sizes. Panagiotakos et al. (2004) observed – in a model adjusted for demographic factors as well as BMI, smoking and other behavioral factors – a 45% lower mean CRP level among those who had studied at the university level as compared to those who had not. Ford et al. (2004) found a borderline association (p = 0.054) among women with less than a high school education, who presented with 0.17 mg/L (SE 0.09) higher *ln*CRP than those with more education. Loucks et al. (2005) observed a similar inverse trend in a multiple linear regression analysis; those with masters or doctoral degrees had a mean CRP (mg/L) level of 3.2 (95% CI 3.0–3.3) whereas those who had not completed high school presented a mean of 4.7 (95% CI 4.5–4.9).

Of the five studies reporting fully adjusted analyses that showed non-significant results, four showed a trend towards an inverse association. Ford et al. (2003) found that men with less than a high school education had double the regression coefficient for *ln*CRP than men with a high school diploma, although this association was not significant (β = 0.132, SE 0.096 and β = 0.065, SE 0.093) when smoking, BMI, alcohol intake and race/ethnicity were included in the model. Bo et al. (2005) found that those with secondary and university educations were protected against elevated CRP levels, but that these associations did not reach significance after adjustment for BMI, physical activity and smoking. Another study showed that those with post-secondary education had mean CRP levels of 1.52 mg/L (SE 0.19) whereas those with less education had 1.97 mg/L (SE 0.10) before body mass index (BMI) and waist-hip ratio (WHR) were included in statistical models, attenuating these associations to the null [45]. Similarly, McDade et al. (2006) found that the 0.06 mg/L reduction in *ln*CRP among those with higher education was made non-significant when variables such as waist circumference and smoking were included in the model.

### Index measures

Eleven studies used index measures, including the British social class system based on occupational status, to create socioeconomic scores or categories [26,38-40,44,45,48,50,51,54,55]. Of the nine studies presenting minimally adjusted results [26,38-40,44,45,48,50,51] – five unadjusted and four adjusted for demographic variables – eight found inverse associations, increasing SEP being associated with lower CRP levels [26,39,40,44,45,48,50,51].

Among the eight studies that investigated this association in multivariable analysis [40,44,45,48,50,51,54,55], five found significantly increasing CRP levels with decreasing SEP [40,44,48,51,54]. These are discussed below.

Mendall et al. (2000) found a significant inverse trend such that father's social class (as a proxy of childhood SEP) of IV (vs. I/II) was associated with a 33% (95% CI 4–69) relative increase in CRP levels among middle-aged men after adjusting for factors including age, BMI, smoking, own social class and alcohol intake. A Finnish study showed that those with low and high SEP had geometric means of CRP of 2.11 and 1.63 mg/L in models adjusted for age, smoking, WHR and prevalent longstanding disease [44]. In a representative sample from the USA, Alley et al. (2005) showed that almost 16% of individuals from families living in poverty had elevated levels of CRP (>10 mg/L), compared to 9% of the remaining subjects. Panagiotakos et al. (2005) reported similar results, with low and high SEP groups being associated with mean CRP levels of 0.21 (SD 0.10) and 0.16 (SD 0.17) mg/L, respectively. This finding remained significant after adjustment for age, sex, smoking, BMI, diet score and physical activity level. St. James O'Reilly (2006) found a significant increase in CRP levels such that in a model adjusted for age, smoking, BMI and use of medications, women, but not men, with a one unit increase in deprivation category had 4.8% (95% CI 0.4–9.5) higher levels of CRP.

Of the three studies using multivariable analyses that showed non-significant results [45,50,55], two observed a trend towards an inverse association [45,50], while a third did not provide details [55], Kivimaki et al. (2005) found that parental and own SEP indicators showed inverse non-significant trends with CRP after including BMI and WHR in statistical models. Rathmann et al. (2006) showed that CRP levels were inversely associated with SEP such that women with low and high SEP had geometric means of 2.17 (SD 2.7) and 1.32 (SD 2.6) mg/L, respectively, which were significant in analyses adjusted for age, smoking, physical activity level and alcohol consumption, but decreased in significance to the borderline level when BMI and WHR were added to the model.

### Income and employment status

Onat et al. (2001) reported that income was inversely associated with CRP levels in minimally adjusted models, among women significantly, but that this association lost significance in fully adjusted models [41], Alley et al. (2005) found that higher income was protective against moderately elevated CRP levels (1.1–3.0 mg/L) when adjusted for variables including age, sex, race/ethnicity, obesity and smoking; effect sizes were not provided.

Four studies reported on variables related to employment status [34,36,43,45]. In minimally adjusted analyses, Mendall et al. (1996) found that father's, but not own occupation was inversely associated with CRP levels in men (effect sizes in next section). Using data from one city in the UK, Danesh et al. (1999) reported a trend in CRP tertiles according to employment status such that 56% of those in the highest CRP tertile were employed compared to 65% employed in the second and 74% in the lowest tertile. In the same study, however, type of job (manual or not) and four other socioeconomic indicators were not associated with CRP in minimally adjusted or fully adjusted models. In minimally adjusted analyses, Ford (2002) found that 57% (SE 1.5) of individuals in the elevated CRP group (>= 85^th ^percentile) had worked during the past two weeks whereas 68% (SE 0.7) in the non-elevated (< 85^th ^percentile) group had worked in the same period. Kivimaki et al. (2005) found that parental, but not own occupation showed inverse trends with CRP but that these associations were not significant in fully adjusted models including anthropometric variables (effect sizes above).

### Early life SEP

Four studies examined early life socioeconomic factors with respect to CRP in adulthood [26,34,45,54]. In minimally adjusted analyses, two British studies found inverse associations between CRP in adulthood and early life SEP. Mendall et al. (1996) found that father's occupation of 75% (SE 5.6) of men in the highest quintile of median CRP was manual, compared to 61% (SE 6.3) in the lowest quintile. Lawlor et al. (2005) found an age-adjusted direct association between CRP and the number of adverse life-course socioeconomic indicators: subjects with two adverse indicators presented a geometric mean CRP level of 1.4 mg/L (SEs not given) compared to 2.2 for those with eight indicators [26]. In fully adjusted models including age, sex, BMI and smoking, two European studies yielded conflicting results. A British study found an inverse association between father's social class and CRP levels in adulthood (effect sizes presented above, Mendall et al., 2000) while a Finnish study found a non-significant inverse trend (effect sizes previous section, Kivimaki et al., 2005) [45,54].

### Race and ethnicity

The second part of this review addressed associations with variables related to race, ethnicity or skin color. Fifteen studies – twelve from the USA, two from the UK and one from Canada – examined CRP levels with respect to such variables [33,35,36,38,40,47,52,53,56-62]. A total of 87285 analyses were represented and some studies used the same sample more than once, as discussed above.

Six presented minimally adjusted results only [33,35,36,38,56,60] and nine [40,47,52,53,57-59,61,62] presented both minimally adjusted (seven unadjusted and two adjusted for demographic variables) and fully adjusted results. Thus, all 15 included studies presented minimally adjusted results, of which only one study failed to report significant associations [53]. Of the nine studies presenting fully adjusted results [40,47,52,53,57-59,61,62], only one failed to show associations [53] and two found that these associations were no longer significant after full adjustment, but that the direction of the effect was maintained [47,61]. The remaining six studies found that the associations remained significant in fully adjusted models.

### NHANES data

Eight of the US studies used data from NHANES data sets [33,35,36,40,52,53,56,60], some with overlapping samples [33,36,56,60,52,53,40]. Seven reports presented minimally adjusted results and all but one [53] showed some significant associations between race/ethnicity and CRP level. Three of these studies [40,52,53] included fully adjusted models, of which two found significant associations [40,52]. All studies presenting significant findings reported higher CRP levels for blacks and Hispanics (or Mexican-Americans) as compared to whites.

In all five studies that reported only minimally adjusted results [33,35,36,56,60], non-whites had significantly higher CRP levels than other groups. In comparing 95^th ^percentiles among elderly white, black and Mexican-Americans, Wener et al. (2000) showed that among men, Mexicans had the highest 95^th ^percentiles (2.59 mg/dL, as presented in Wener et al., 2000; 95% CI not available due to missing information), followed by blacks (2.40 mg/dL) and then whites (1.24 mg/dL, 95% CI 0.84–1.64), with women following the same pattern. Wong et al. (2001) presented similar results, showing that the mean (SD) CRP levels among white, black and Mexican men were 0.37 (0.52), 0.47 (0.75) and 0.39 (0.65) mg/dL; and 0.46 (0.62), 0.61 (0.86) and 0.64 (1.32) mg/dL among women. Ford (2002) showed that whites composed 69% (SE 1.9) and 78% (SE 1.3) of the >= 85^th ^and <85^th ^percentile of CRP levels, respectively. Abramson et al. (2002) found that African-Americans had 1.75 higher odds (calculated from given distributions; no 95% CI available) of being in the elevated CRP group (>= 0.66 mg/dL) as compared to whites.

Danner et al. (2003) found African – and Mexican-Americans at higher risk for elevated CRP (>= 0.22 mg/dL) as compared to whites. The highest prevalence ratio (PR) compared to whites of the same sex was among African-American men (PR = 1.57), followed by African – and Mexican-American women (PR = 1.44) and Mexican-American men (prevalence ratio = 1.24, compared to white individuals of same sex, calculated from given distributions; no 95% CI available).

Conflicting results were found in the three studies that utilized fully adjusted models including age, sex, smoking and BMI among individuals aged at least 20 years. Ford et al. (2003) observed no associations between race/ethnicity and CRP levels among men. In a 2004 study, the same author reported that Mexican-American women had on average 0.29 mg/L (SE 0.07) higher lnCRP levels than their white counterparts [52]. Alley et al. (2005) found that blacks had significantly higher odds of being in either the high (OR = 1.45 95% CI: 1.16–1.80) or very high (OR = 2.32 95% CI: 1.76–3.08) CRP groups as compared to other groups in fully adjusted models including demographic and lifestyle variables and BMI [40].

### Other studies from North America and the UK

Four studies from the USA, which did not use NHANES data, were included. One found median CRP levels of 3.0 mg/L in blacks and 2.3 in whites of both sexes, which remained significant after adjusting for traditional cardiovascular risk factors [62]. In the same study, black men and women made up a significantly larger proportion of the high risk CRP group (>3 mg/L) than white men. Similarly, Matthews et al. (2005) used a multi-community sample and found that after adjusting for variables such as education, physical activity and % calories from fat, African-American, Hispanic and white women presented median (IQR) CRP levels of 3.0 (1.0–7.2), 2.3 (1.0–5.1) and 1.4 (0.6–3.9), respectively [58]. A small study among older individuals found no associations between blacks and whites, nor between Latinos and whites [47]. Another study using samples from multiple communities across the USA found that Hispanic men and women had the highest levels of CRP (2.51, 3.39, respectively), followed by African-Americans (2.12, 3.19), Caucasians (3.20, 2.75) and Chinese (0.95, 1.20), after adjusting for covariates including age, BMI, smoking, physical activity and estrogen medications [57]. Significance testing was not done between ethnic groups.

Both studies from the UK found higher CRP levels among South Asians compared to European whites in minimally adjusted analyses [38,61]. Forouhi et al. (2001) observed that the median CRP level among South Asian women was 1.35 (95% CI 0.72–3.04) compared to half that level, 0.70 mg/L (95% CI 0.41–1.7), among European women. This unadjusted association was significant; fully adjusted models were not used. In another UK study Chambers et al. (2001) showed that the age-adjusted association among the same ethnic groups lost significance after adjustment for age, BMI and smoking, but the direction of effect was maintained. The Canadian study found significant differences between ethnic groups [59]. In fully adjusted models including BMI as a covariate, Chinese, European, South Asian and Aboriginals had mean CRP levels of 1.72 (SE 0.13), 2.13 (SE 0.12), 2.72 (SE 0.12) and 2.85 (SE 0.15) mg/L, respectively, with non-significant differences between European and Chinese, and between South Asians and Aboriginals.

## Discussion

The overwhelming majority of the 32 studies reported inverse associations between CRP levels and SEP and significant differences among racial/ethnic groups, even after controlling for possible confounding and mediating variables. Individuals with African, Latin American or South Asian ancestry had higher levels than those with European background. Both sets of findings are consistent with previous studies showing similar associations between other CVD risk factors and SEP and race/ethnicity [3,4,8-10,63]. Although the notion that CRP levels are causally associated with CVD has been challenged by Mendelian Randomization studies, there is little question that CRP constitutes a reliable marker for low-grade inflammation that identifies high-risk individuals [26,29].

Some strengths and limitations of this review warrant mention. It included studies using population-based studies, which minimizes selection bias. On the other hand, studies were largely from in high-income countries, notably from the USA and several European countries. The only low/middle-income country represented was Turkey; Onat et al. (2001) found inconsistent results. Further studies from low and middle-income countries are required to investigate whether the results from this review are also applicable to these regions. It is worth noting that some studies excluded individuals with CRP levels >10 mg/L (the cut-off for acute inflammation), which would make their results applicable only to low-level inflammation.

When interpreting adjusted results, we gave special emphasis to differentiating possible confounders from mediating variables, based on a conceptual model (Figure 1). In examining the association between SEP and CRP levels, adjustment for variables in level 1 of the model (age, sex and race/ethnicity, in addition to genetic factors) should account for confounding. None of the present studies adjusted for genetic markers, and these would only confound the association if they were unequally distributed among SEP groups. Also, several samples were ethnically homogeneous, rendering adjustment for this variable unnecessary. Therefore, for these studies adjustment for age and sex should take care of the issue of known and measurable confounders.

Of the 20 studies presenting minimally adjusted results, 13 presented unadjusted analyses and seven presented results adjusted for demographic factors. All of the latter showed significant inverse associations between SEP and CRP.

The fully adjusted models included age and sex in all studies, BMI in all but one (Matthews et al. 2005 used %kcal from fat) and smoking in all but one study [59]. Nine of 21 studies did not adjust for hormone replacement therapy (HRT) use [40,41,46-51,59]. Thus, five common variables were adjusted for in most studies (age, sex, BMI, smoking and HRT). Therefore, the fully adjusted model in nearly every study included both confounding (age, sex) and potentially mediating (BMI, smoking, HRT) variables. These analyses answer the question "what is the effect of SEP on CRP levels that does not pass through the mediating factors that are in the model?", not the question of the overall effect of SEP.

The persistence of significant associations in most fully adjusted analyses suggest that low SEP is a risk factor for elevated CRP levels (although not necessarily cardiovascular risk), even when some of the potential mediators of this association are controlled for. It also suggests that there are other pathways by which SEP may affect CRP levels [30]. For example, it has been postulated that high levels of stress accumulated throughout the life-course, which is often the case among the poor and disadvantaged, may adversely impact health outcomes [64,65].

Socioeconomic status in childhood, independently of adult socioeconomic conditions, has been shown to be associated with mortality among adults, indicating specific effects of early deprivation [66]. Four studies included in this review suggest a similar association between early SEP and CRP levels. The only exception was a high-quality Finnish study which reported a significant association in age and sex-adjusted analyses that lost significance after adjustment for BMI and WHR, that according to our framework represent possible mediating factors [45]. It is likely that adverse features are programmed in intrauterine and early life under conditions of poor SEP during physiologically plastic periods and track into adulthood, manifesting as increased risk for disease, regardless of later improvement of socioeconomic conditions [64,67,68].

The findings on racial/ethnic groups are now addressed. The range of ethnicities represented in the studies was relatively limited. In studies from the UK, only European whites and South Asians comprised the study samples, whereas in studies from the USA, four general categories were presented (white, black/African-American, Hispanic/Mexican-American and other). Canadian studies were more diverse, including Aboriginals, Europeans and at least two Asian groups (eg: Chinese, Japanese, South Asian).

In terms of the conceptual model (Figure 1), the effects of race/ethnicity on CRP levels may be confounded by other level 1 variables (age, sex and genetic factors) [69]. Because non-whites tend to have poorer education and income than whites in North America and Europe, SEP can legitimately be considered a potential mediating factor in the association between ethnicity and health-related outcomes [70-72]. Models that adjust for SEP, therefore, are answering the question on what the effect of race/ethnicity is on CRP that is not mediated by SEP. Models that further adjust for level 3 variables (such as BMI, smoking, etc) are answering the question of what effect of race/ethnicity remains outside these pathways.

Both in the minimally and fully adjusted models, findings on the association between race/ethnicity and CRP were highly consistent. In studies from North America, blacks and Hispanics tended to have higher CRP than whites, and individuals of East Asian descent (Chinese, Japanese) tended to have the lowest levels. In UK studies, South Asians were shown to have higher levels of CRP than European whites. The high CVD rates among black Americans and South Asians in Europe are consistent with these findings on CRP [9].

## Conclusion

Socioeconomic status was independently and inversely associated with CRP levels among adults in several high-income countries. Studies regarding the effects of early life and adult SEP on risk factors such as CRP are still needed, especially from low and middle-income countries.

Race/ethnicity was independently associated with CRP levels such that those of African, Latin or South Asian descent were at higher risk for elevated CRP than subjects of European descent. Given the complex inter-relationships between exposures, confounding and mediating variables, racial/ethnic determinants of disease can be difficult to study and interpret [73]. Nevertheless, collaborative studies from multiple countries may lead to increased insight regarding the effects of these variables on risk factors and disease outcomes.

A full understanding of the associations between SEP and race/ethnicity on the one hand, and CRP on the other, was precluded by the fact that nearly all studies included statistical adjustment for variables that are likely mediating factors in this causal pathway. The fact that most studies show significant results despite such over adjustment is reassuring, but the true magnitude of these associations is being underestimated. Epidemiological studies addressing what has been described as the "causes of causes", that is, the distal determinants of health and disease, would greatly profit from the use of conceptual models spelling out causal pathways that allow to differentiate confounding from mediating factors [74,75].

These findings indicate that poorer, non-white individuals are the highest risk for elevated CRP levels. This is consistent with previous data showing that these groups have elevated incidence and prevalence of almost all disease states. Scientists and policy makers should capitalize upon results from the host of studies that have already confirmed such associations in attempts to curb the growing incidence of chronic disease caused by low SEP and ever-increasing socioeconomic gaps [76].

## Competing interests

The author(s) declare that they have no competing interests.

## Authors' contributions

AN carried out the literature search and wrote the article with CV. Both authors read and approved the final manuscript.

## Pre-publication history

The pre-publication history for this paper can be accessed here:



## Supplementary Material

Additional file 1 Population-based studies examining socioeconomic factors or race/ethnicity as independent and C-reactive protein (CRP) level as dependent variables. The data provided represent the findings from a systematic review of population-based studies published through December 2006 that examined C-reactive protein levels in adults in relation to socioeconomic factors or race/ethnicity. Click here for file

Additional file 2 Summary of results and effect sizes for the associations between socioeconomic factors and race/ethnicity with C-reactive protein (CRP) levels. The data provided summarize the effect sizes of the associations between C-reactive protein levels in adults in relation to socioeconomic factors or race/ethnicity from a systematic review of population-based studies. Click here for file

## References

[B1] Lopez AD, Mathers CD, Ezzati M, Jamison DT, Murray CJL (2006). Global Burden of Disease and Risk Factors.

[B2] WHO WHO Global Strategy on Diet, Physical Activity and Health. Cardiovascular disease: prevention and control. http://www.who.int/dietphysicalactivity/publications/facts/cvd/en/.

[B3] Cooper R, Cutler J, Desvigne-Nickens P, Fortmann SP, Friedman L, Havlik R, Hogelin G, Marler J, McGovern P, Morosco G, Mosca L, Pearson T, Stamler J, Stryer D, Thom T (2000). Trends and Disparities in Coronary Heart Disease, Stroke, and Other Cardiovascular Diseases in the United States : Findings of the National Conference on Cardiovascular Disease Prevention. Circulation.

[B4] Cooper RS (2001). Social inequality, ethnicity and cardiovascular disease. Int J Epidemiol.

[B5] Dalstra JAA, Kunst AE, Borrell C, Breeze E, Cambois E, Costa G, Geurts JJM, Lahelma E, Van Oyen H, Rasmussen NK, Regidor E, Spadea T, Mackenbach JP (2005). Socioeconomic differences in the prevalence of common chronic diseases: an overview of eight European countries. Int J Epidemiol.

[B6] Mackenbach JP, Cavelaars AEJM, Kunst AE, Groenhof F (2000). Socioeconomic inequalities in cardiovascular disease mortality. An international study. Eur Heart J.

[B7] Winkleby MA, Kraemer HC, Ahn DK, Varady AN (1998). Ethnic and Socioeconomic Differences in Cardiovascular Disease Risk Factors: Findings for Women From the Third National Health and Nutrition Examination Survey, 1988-1994. JAMA.

[B8] Yarnell J, Yu S, McCrum E, Arveiler D, Hass B, Dallongeville J, Montaye M, Amouyel P, Ferrieres J, Ruidavets JB, Evans A, Bingham A, Ducimetiere P, for the P (2005). Education, socioeconomic and lifestyle factors, and risk of coronary heart disease: the PRIME Study. Int J Epidemiol.

[B9] Anand SS, Yusuf S, Vuksan V, Devanesen S, Teo KK, Montague PA, Kelemen L, Yi C, Lonn E, Gerstein H (2000). Differences in risk factors, atherosclerosis, and cardiovascular disease between ethnic groups in Canada: the Study of Health Assessment and Risk in Ethnic groups (SHARE). Lancet.

[B10] Sundquist J, Winkleby MA, Pudaric S (2001). Cardiovascular Disease Risk Factors Among Older Black, Mexican-American, and White Women and Men: An Analysis of NHANES III, 1988-1994. J Am Geriatr Soc.

[B11] Ross R (1999). Mechanisms of Disease: Atherosclerosis - An Inflammatory Disease. N Engl J Med.

[B12] Pepys MB, Hirschfield GM (2003). C-reactive protein: a critical update. J Clin Invest.

[B13] Ridker PM, Cushman M, Stampfer MJ, Tracy RP, Hennekens CH (1997). Inflammation, Aspirin, and the Risk of Cardiovascular Disease in Apparently Healthy Men. N Engl J Med.

[B14] Koenig W, Sund M, Fröhlich M, Fischer HG, Löwel H, Döring A, Hutchinson WL, Pepys MB (1999). C-Reactive Protein, a Sensitive Marker of Inflammation, Predicts Future Risk of Coronary Heart Disease in Initially Healthy Middle-Aged Men. Results From the MONICA (Monitoring Trends and Determinants in Cardiovascular Disease) Augsburg Cohort Study, 1984 to 1992. Circulation.

[B15] Danesh J, Wheeler JG, Hirschfield GM, Eda S, Eda G, Rumley A, Lowe GDO, Pepys MB, Gudnason V (2004). C-Reactive Protein and Other Circulating Markers of Inflammation in the Prediction of Coronary Heart Disease. N Engl J Med.

[B16] Ridker PM, Buring JE, Shih J, Matias M, Hennekens CH (1998). Prospective Study of C-Reactive Protein and the Risk of Future Cardiovascular Events Among Apparently Healthy Women. Circulation.

[B17] Cesari M, Penninx BW, Newman AB, Kritchevsky SB, Nicklas BJ, Sutton-Tyrrell K, Rubin SM, Ding J, Simonsick EM, Harris TB, Pahor M (2003). Inflammatory markers and onset of cardiovascular events: results from the Health ABC study.. Circulation.

[B18] Freeman DJ, Norrie J, Caslake MJ, Gaw A, Ford I, Lowe GDO, O'Reilly DSJ, Packard CJ, Sattar N (2002). C-Reactive Protein Is an Independent Predictor of Risk for the Development of Diabetes in the West of Scotland Coronary Prevention Study. Diabetes.

[B19] Hu FB, Meigs JB, Li TY, Rifai N, Manson JAE (2004). Inflammatory Markers and Risk of Developing Type 2 Diabetes in Women. Diabetes.

[B20] Thorand B, Lowel H, Schneider A, Kolb H, Meisinger C, Frohlich M, Koenig W (2003). C-Reactive Protein as a Predictor for Incident Diabetes Mellitus Among Middle-aged Men: Results From the MONICA Augsburg Cohort Study, 1984-1998. Arch Intern Med.

[B21] Ridker PM, Glynn RJ, Hennekens CH (1998). C-Reactive Protein Adds to the Predictive Value of Total and HDL Cholesterol in Determining Risk of First Myocardial Infarction. Circulation.

[B22] Blake GJ, Rifai N, Buring JE, Ridker PM (2003). Blood Pressure, C-Reactive Protein, and Risk of Future Cardiovascular Events. Circulation.

[B23] Pasceri V, Willerson JT, Yeh ETH (2000). Direct Proinflammatory Effect of C-Reactive Protein on Human Endothelial Cells. Circulation.

[B24] Verma S, Szmitko PE, Yeh ETH (2004). C-Reactive Protein: Structure Affects Function. Circulation.

[B25] Pepys MB (2005). CRP or not CRP? That Is the Question. Arterioscler Thromb Vasc Biol.

[B26] Lawlor DA, Davey Smith G, Rumley A, Lowe GD, Ebrahim S (2005). Associations of fibrinogen and C-reactive protein with prevalent and incident coronary heart disease are attenuated by adjustment for confounding factors. British Women's Heart and Health Study. Thromb Haemost.

[B27] Davey Smith G, Lawlor DA, Harbord R, Timpson N, Rumley A, Lowe GDO, Day INM, Ebrahim S (2005). Association of C-Reactive Protein With Blood Pressure and Hypertension: Life Course Confounding and Mendelian Randomization Tests of Causality. Arterioscler Thromb Vasc Biol.

[B28] Timpson NJ, Lawlor DA, Harbord RM, Gaunt TR, Day INM, Palmer LJ, Hattersley AT, Ebrahim S, Lowe DOG, Rumley A, Davey-Smith G (2005). C-reactive protein and its role in metabolic syndrome: mendelian randomisation study. Lancet.

[B29] Casas JP, Shah T, Cooper J, Hawe E, McMahon AD, Gaffney D, Packard CJ, O'Reilly DS, Juhan-Vague I, Yudkin JS, Tremoli E, Margaglione M, Di Minno G, Hamsten A, Kooistra T, Stephens JW, Hurel SJ, Livingstone S, Colhoun HM, Miller GJ, Bautista LE, Meade T, Sattar N, Humphries SE, Hingorani AD (2006). Insight into the nature of the CRP-coronary event association using Mendelian randomization. Int J Epidemiol.

[B30] Victora CG, Huttly SR, Fuchs SC, Olinto MTA (1997). The Role of Conceptual Frameworks in Epidemiological Analysis: A Hierarchical Approach. Int J Epidemiol.

[B31] Oakes M, Kaufman J (2005). Methods for social epidemiology.

[B32] Moher D, Cook DJ, Eastwood S, Olkin I, Rennie D, Stroup DF, for the QUOROM Group (1999). Improving the quality of reports of meta-analyses of randomised controlled trials: the QUOROM statement. Lancet.

[B33] Abramson JL, Weintraub WS, Vaccarino V (2002). Association Between Pulse Pressure and C-Reactive Protein Among Apparently Healthy US Adults. Hypertension.

[B34] Mendall MA, Patel P, Ballam L, Strachan D, Northfield TC (1996). C reactive protein and its relation to cardiovascular risk factors: a population based cross sectional study. BMJ.

[B35] Danner M, Kasl SV, Abramson JL, Vaccarino V (2003). Association Between Depression and Elevated C-Reactive Protein. Psychosom Med.

[B36] Ford ES (2002). Does exercise reduce inflammation? Physical activity and C-reactive protein among U.S. adults.. Epidemiology.

[B37] Ford ES, Giles WH (2000). Serum C-Reactive Protein and Self-Reported Stroke : Findings From the Third National Health and Nutrition Examination Survey. Arterioscler Thromb Vasc Biol.

[B38] Forouhi NG, Sattar N, McKeigue PM (2001). Relation of C-reactive protein to body fat distribution and features of the metabolic syndrome in Europeans and South Asians. Int J Obes Relat Metab Disord.

[B39] Sattar N, McConnachie A, O'Reilly D, Upton MN, Greer IA, Davey Smith G, Watt G (2004). Inverse association between birth weight and C-reactive protein concentrations in the MIDSPAN Family Study. Arterioscler Thromb Vasc Biol.

[B40] Alley DE, Seeman TE, Ki Kim J, Karlamangla A, Hu P, Crimmins EM (2005). Socioeconomic status and C-reactive protein levels in the US population: NHANES IV. Brain Behav Immun.

[B41] Onat A, Sansoy V, B BY, Keles I, Uysal O, Hergenc G (2001). C-reactive protein and coronary heart disease in western Turkey. Am J Cardiol 2001.

[B42] Bo S, Gentile L, Ciccone G, Baldi C, Benini L, Dusio F, Lucia C, Forastiere G, Nuti C, Cassader M, Franco Pagano G (2005). The metabolic syndrome and high C-reactive protein: prevalence and differences by sex in a southern-European population-based cohort. Diabetes Metab Res Rev.

[B43] Danesh J, Muir J, Wong Y, Ward M, Gallimore JR, Pepys MB (1999). Risk factors for coronary heart disease and acute-phase proteins. A population-based study. Eur Heart J.

[B44] Jousilahti P, Salomaa V, Rasi V, Vahtera E, Palosuo T (2003). Association of markers of systemic inflammation, C reactive protein, serum amyloid A, and fibrinogen, with socioeconomic status. J Epidemiol Commun H.

[B45] Kivimaki M, Lawlor DA, Juonala M, Smith GD, Elovainio M, Keltikangas-Jarvinen L, Vahtera J, Viikari JS, Raitakari OT (2005). Lifecourse socioeconomic position, C-reactive protein, and carotid intima-media thickness in young adults: the cardiovascular risk in Young Finns Study. Arterioscler Thromb Vasc Biol.

[B46] Loucks EB, Sullivan LM, Hayes LJ, D'Agostino RB, Larson MG, Vasan RS, Benjamin EJ, Berkman LF (2006). Association of Educational Level with Inflammatory Markers in the Framingham Offspring Study. Am J Epidemiol.

[B47] McDade TW, Hawkley LC, Cacioppo JT (2006). Psychosocial and behavioral predictors of inflammation in middle-aged and older adults: the Chicago health, aging, and social relations study. Psychosom Med.

[B48] Panagiotakos DB, Pitsavos C, Manios Y, Polychronopoulos E, Chrysohoou CA, Stefanadis C (2005). Socio-economic status in relation to risk factors associated with cardiovascular disease, in healthy individuals from the ATTICA study. Eur J Cardiovasc Prev Rehabil.

[B49] Panagiotakos D, Pitsavos C, Chrysohoou C, Skoumas J, Toutouza M, Belegrinos D, Toutouzas P, Stefanadis C (2004). The association between educational status and risk factors related to cardiovascular disease in healthy individuals: The ATTICA study. Ann Epidemiol.

[B50] Rathmann W, Haastert B, Giani G, Koenig W, Imhof A, Herder C, Holle R, Mielck A (2006). Is inflammation a causal chain between low socioeconomic status and type 2 diabetes? Results from the KORA Survey 2000. Eur J Epidemiol.

[B51] St J O'Reilly D, Upton MN, Caslake MJ, Robertson M, Norrie J, McConnachie A, Watt GCM, Packard CJ, on behalf of the Midspan and Woscops study groups (2006). Plasma C reactive protein concentration indicates a direct relation between systemic inflammation and social deprivation. Heart.

[B52] Ford ES, Giles WH, Mokdad AH, Myers GL (2004). Distribution and Correlates of C-Reactive Protein Concentrations among Adult US Women. Clin Chem.

[B53] Ford ES, Giles WH, Myers GL, Mannino DM (2003). Population Distribution of High-Sensitivity C-reactive Protein among US Men: Findings from National Health and Nutrition Examination Survey 1999-2000. Clin Chem.

[B54] Mendall MA, Strachan DP, Butland BK, Ballam L, Morris J, Sweetnam PM, Elwood PC (2000). C-reactive protein: relation to total mortality, cardiovascular mortality and cardiovascular risk factors in men. Eur Heart J.

[B55] Williams MJ, Williams SM, Milne BJ, Hancox RJ, Poulton R (2004). Association between C-reactive protein, metabolic cardiovascular risk factors, obesity and oral contraceptive use in young adults. Int J Obes Relat Metab Disord.

[B56] Wong ND, Pio J, Valencia R (2001). Distribution of C-Reactive Protein and Its Relation to Risk Factors and Coronary Heart Disease Risk Estimation in the National Health and Nutrition Examination Survey (NHANES) III. Prev Cardiol.

[B57] Lakoski SG, Cushman M, Criqui M, Rundek T, Blumenthal RS, D'Agostino Jr RB, Herrington DM (2006). Gender and C-reactive protein: data from the Multiethnic Study of Atherosclerosis (MESA) cohort. Am Heart J.

[B58] Matthews KA, Sowers MF, Derby CA, Stein E, Miracle-McMahill H, Crawford SL, Pasternak RC (2005). Ethnic differences in cardiovascular risk factor burden among middle-aged women: Study of Women's Health Across the Nation (SWAN). Am Heart J.

[B59] Anand SS, Razak F, Yi Q, Davis B, Jacobs R, Vuksan V, Lonn E, Teo K, McQueen M, Yusuf S (2004). C-Reactive Protein as a Screening Test for Cardiovascular Risk in a Multiethnic Population. Arterioscler Thromb Vasc Biol.

[B60] Wener MH, Daum PR, McQuillan GM (2000). The influence of age, sex, and race on the upper reference limit of serum C-reactive protein concentration. J Rheumatol.

[B61] Chambers JC, Eda S, Bassett P, Karim Y, Thompson SG, Gallimore JR, Pepys MB, Kooner JS (2001). C-Reactive Protein, Insulin Resistance, Central Obesity, and Coronary Heart Disease Risk in Indian Asians From the United Kingdom Compared With European Whites. Circulation.

[B62] Khera A, McGuire DK, Murphy SA, Stanek HG, Das SR, Vongpatanasin W, Wians Jr FH, Grundy SM, de Lemos JA (2005). Race and gender differences in C-reactive protein levels. J Am Coll Cardiol.

[B63] Silventoinen K, Pankow J, Jousilahti P, Hu G, Tuomilehto J (2005). Educational inequalities in the metabolic syndrome and coronary heart disease among middle-aged men and women. Int J Epidemiol.

[B64] Gillman MW (2002). Epidemiological challenges in studying the fetal origins of adult chronic disease. Int J Epidemiol.

[B65] Singh-Manoux A, Ferrie JE, Chandola T, Marmot M (2004). Socioeconomic trajectories across the life course and health outcomes in midlife: evidence for the accumulation hypothesis?. Int J Epidemiol.

[B66] Davey Smith G, Hart C, Blane D, Hole D (1998). Adverse socioeconomic conditions in childhood and cause specific adult mortality: prospective observational study. BMJ.

[B67] Godfrey K, Barker DJP (2001). Fetal programming and adult health. Public Health Nutrition.

[B68] Lucas A, Fewtrell MS, Cole TJ (1999). Fetal origins of adult disease-the hypothesis revisited. BMJ.

[B69] Milyo J, Mellor JM (2003). On the Importance of Age-Adjustment Methods in Ecological Studies of Social Determinants of Mortality. Health Serv Res.

[B70] Freeman H (1991). Race, Poverty, and Cancer. J Natl Cancer Inst.

[B71] US Census Bureau Historical poverty tables. http://www.census.gov/hhes/www/poverty/poverty.html.

[B72] Joseph Rowntree Foundation Ethnic groups and low income distribution. http://www.jrf.org.uk/.

[B73] Lynch J, Davey Smith G, Harper S, Hillemeier M, Ross N, Kaplan GA, Wolfson M (2004). Is income inequality a determinant of population health? Part I. A systematic review.. The Millbank Quarterly.

[B74] Krieger N (2001). Theories for social epidemiology in the 21st century: an ecosocial perspective. Int J Epidemiol.

[B75] Wilkinson RG, Marmot MG (2006). Social Determinants of Health.

[B76] Blakely T, Hales S, Kieft C, Wilson N, Woodward A (2005). The global distribution of risk factors by poverty level. Bull World Health Organ.

