# Supplementary material for: Socioeconomic and racial/ethnic differentials of C-reactive protein levels: a systematic review of population-based studies
Source: BMC Public Health. 2007 Aug 17;7:212. doi: 10.1186/1471-2458-7-212 (PMC2018719; doi:10.1186/1471-2458-7-212)
Supplement: Additional file 1 — Population-based studies examining socioeconomic factors or race/ethnicity as independent and C-reactive protein (CRP) level as dependent variables. The data provided represent the findings from a systematic review of population-based studies published through December 2006 that examined C-reactive protein levels in adults in relation to socioeconomic factors or race/ethnicity. [file 1471-2458-7-212-S1.pdf]

Table 1: Population-based studies examining socioeconomic factors or race/ethnicity as independent and C-reactive protein (CRP) level as dependent variables.

[Author, year]<br>Sample population, study type, sampling method	N	Age, mean or range (y)	Minimally-adjusted and fully adjusted association with independent variables[**]<br>Socioeconomic factors	 Race/ethnicity[#]	Treatment of dependent variable[##] and observations
[Abramson, 2002][*]<br>US national sample[a], cross-sectional, stratified multi-stage probability sample	9867	>= 17	(-) Education	(+) African-American vs. White and "Other" categories	Dichotomous (< or >= 0.66 mg/dL)
[Alley, 2005]<br>US national sample[a], cross-sectional, stratified multi-stage probability sample	6830	>= 20	(-) Above poverty line	(+) Black<br>(+) Hispanic<br>(n/s) Other<br>White reference category<br>At CRP 3.1-10.0 mg/L	Categorical (<=1.0, 1.1-3.0, 3.1-10, >10.0 mg/L CRP),<br><br>Significant associations at age <=80 y
			(-) Above poverty line<br>(-) Education<br>(-) Household income<br>At CRP>10 mg/L	(+) Black, at CRP>3 mg/L<br>(n/s) Hispanic<br>(n/s) Other<br>White reference category	
[Anand, 2004]<br>Four communities in Canada[b], cross-sectional, random sample of age-eligible	1250	35-75	--	(+) Lowest to highest: Chinese, European, South Asian, Aboriginal	Continuous
				(+) Lowest to highest: Chinese, European, South Asian, Aboriginal<br>(n/s) European vs. Chinese<br>(n/s) South Asian vs. Aboriginal	
[Bo, 2005]<br>Asti province, Italy, cross-sectional, all age-eligible population from 6 GPs representative of districts in province	1650	45-64	(-) Education	--	Dichotomous (< or >= 3.0 mg/L)
			(n/s) Education		

[Chambers, 2001]<br>London, UK, cross-sectional, random sample of Indian Asians and European Whites from 56 GPs	1025	35-60,<br>men	--	(+) Indian Asian<br>European White reference category<br>Age adjusted	Continuous
				(n/s) Indian Asian<br>European White reference category	
[Danesh, 1999]<br>Bedfordshire, UK, cross-sectional, randomly selected control group for trial study from five GPs	704	35-64	(-) Employment<br>(n/s) Completed education by age 16 y<br>(n/s) In rented housing<br>(n/s) Car ownership<br>(n/s) Manual worker<br>(n/s) Marital status	--	Categorical (thirds of CRP)
			(-) Employment<br>(n/s) Completed education by age 16 y<br>(n/s) In rented housing<br>(n/s) Car ownership<br>(n/s) Manual worker<br>(n/s) Marital status		
[Danner, 2003][*]<br>US national sample[a], cross-sectional, stratified multi-stage probability sample	6149	17-39	(-) Education (women)	(+) Lowest to highest (men): Mexican-American, “Other”, African-American<br>(+) Lowest to highest (women): “Other”, Mexican-American / African-American<br>White reference categories	Dichotomous (< or >= 0.22 mg/dL)
[Ford, 2000][*]<br>US national sample[a], cross-sectional, stratified multi-stage probability sample	8850	>= 40	(-) Education	--	Categorical (<= 0.21, > 0.21-< 0.55, >= 0.55 mg/dL)
[Ford, 2002][*]<br>US national sample[a], cross-sectional, stratified multi-stage probability sample	13748	>= 20	(-) Recently employed<br>(n/s) Education<br>Age adjusted	(+) Non-white<br>Age adjusted	Dichotomous (< or >= 85th percentile)

[Ford, 2003]<br>US national sample[a], cross-sectional, stratified multi-stage probability sample	1940	>= 20, men	Not presented	(n/s) Mexican-American<br>(n/s) African-American<br>(n/s) "Other"<br>White reference category	Continuous
			(n/s) Education	(n/s) Mexican-American<br>(n/s) African-American<br>(n/s) "Other"<br>White reference category	
[Ford, 2004]<br>US national sample[a], cross-sectional, stratified multi-stage probability sample	1912	>= 20, women	Not presented	(+) Lowest to highest: White, African-American, Mexican-American	Continuous
			(-) Education (<high-school group)	(+) Mexican-American<br>(n/s) African-American<br>(n/s) "Other"<br>White reference category	
[Forouhi, 2001][*]<br>London, UK, cross-sectional, random sample of South Asians and Europeans from four GPs	113	40-55	(n/s) Social class	(+) South Asian<br>Among women<br>European White reference category	Continuous
[Jousilahti, 2003]<br>Eastern and southern Finland[c], cross-sectional, stratified random sample of men	1503	45-74, men	(-) SES determined by education and total family income	--	Continuous,<br><br>Association significant in <60 y groups
			(-) SES determined by education and total family income		
[Khera, 2005]<br>Dallas County, Texas, USA, cross-sectional, probability-based random sample	2749	30-65	--	(+) Black women<br>White reference category	Continuous and categorical
				(+) Black women<br>(n/s) Black men<br>White men reference category	

[Kivimaki, 2005]<br>Finnish national sample, cross-sectional and prospective analysis within prospective cohort[d], random sample of national register	2290	24-39	(-) Parental occupation<br>(n/s) Parental education<br>(n/s) Own occupation<br>(-) Own education<br>Age and sex adjusted<br>―<br>(n/s) Parental occupation<br>(n/s) Parental education<br>(n/s) Own occupation<br>(n/s) Own education	--	Continuous
[Lakoski, 2006]<br>Six communities in the USA[e], cross-sectional analysis within prospective cohort, stratified random, geographic or random digit dialing of age and race-eligible	6814	45-84	--	(+) Lowest to highest (men): Chinese, Caucasian, African-American, Hispanic<br>(+) Lowest to highest (women): Chinese, Caucasian, Hispanic, African-American<br>―<br>(+) Lowest to highest: Chinese, Caucasian, African-American, Hispanic	Continuous<br><br>Racially stratified analysis of gender; no significance tests between ethnic groups
[Lawlor, 2005][*]<br>UK, cross-sectional analysis within prospective cohort[f], random sample	3745	60-79, women	(-) Life-course SES, determined by index<br>Age adjusted	--	Categorical (quartiles of CRP)
[Loucks, 2006]<br>Framingham, Massachusetts, USA, cross-sectional analysis within prospective cohort, offspring or spouse of offspring of Framingham Heart Study (random sample of two-thirds of adult population of Framingham)	2729	62.1, mean	(-) Education<br>Age and sex adjusted<br>―<br>(-) Education	--	Continuous

[Matthews, 2005]<br>Seven sites in the USA[g], cross-sectional analysis within prospective cohort, random digit-dialing	2834	42-52	--	(+) Lowest to highest: Japanese, Chinese, White, Hispanic, African-American<br>---<br>(+) Lowest to highest: Japanese, Chinese, White, Hispanic, African-American<br>(n/s) African-American vs. Hispanic<br>(n/s) Hispanic vs. White	Continuous
[McDade, 2006]<br>Cook County, Illinois, USA, cross-sectional analysis within sub-sample of prospective cohort[h], multi-stage probability sample of age-eligible	173	50-67	(-) Education<br>Age, gender and ethnicity adjusted<br>---<br>(n/s) Education	(+) Lowest to highest: European-American, Latino-American, African-American<br>---<br>(n/s) African-American<br>(n/s) Latino-American<br>European-American reference category (n=153)	Continuous
[Mendall, 1996][*]<br>London, UK, cross-sectional, random sample of Whites from GPs in Merton, Sutton, and Wandsworth District Health Authorities	303	50-69, men	(-) Father's occupation not manual<br>(n/s) Own occupation not manual	--	Categorical (fifths of CRP)
[Mendall, 2000]<br>Caerphilly and 5 villages in South Wales, cross-sectional and prospective analysis within prospective cohort[i], 100% of age-eligible men	1395	45-59, men	Not presented<br>---<br>(-) Childhood SES, determined by father's social class<br>(n/s) Current social class	--	Continuous

[Onat, 2001]<br>Three regions of Western Turkey, cross-sectional analysis within sub-sample of prospective cohort[j], stratified random sample	1046	>= 30	(-) Family income<br>Among women<br>(n/s) Family income (n=690)	--	Continuous
[Panagiotakos, 2004]<br>Attica province, Greece, cross-sectional, multi-stage random sample[k]	2271	> 18	(-) SES, determined by formal education level<br>(-) SES, determined by formal education level	--	Continuous
[Panagiotakos, 2005]<br>Attica province, Greece, cross-sectional, multi-stage random sample[k]	3042	> 18	(-) Lowest to highest, by SES tertile: 2nd, 3rd, 1st<br>(-) SES, determined by family income and education	--	Continuous
[Rathmann, 2006]<br>Augsburg region, Germany, cross-sectional, cluster sample of region[l]	1653	55-74	(-) SES, determined by occupation; education; vocational training and income (women)<br>Age adjusted<br>(n/s) SES, determined by occupation, education, vocational training and income	--	Continuous
[Sattar, 2004][*]<br>Two Scottish towns[m], cross-sectional analysis within prospective cohort, offspring of original cohort (all aged 45-64 y population)	1663	30-59	(-) SES, deprivation index, determined by postcode of residence	--	Continuous

[St J O'Reilly, 2006]<br>Two Scottish towns[m], cross-sectional analysis within prospective cohort, offspring of original cohort (all aged 45-64 y population)	2101	30-59	(-) SES, deprivation index, determined by postcode of residence<br>Age adjusted	--	Continuous
			(-) SES, deprivation index, determined by postcode of residence (women)		
[Thorand, 2003][*]<br>Augsburg region, Germany, cross-sectional, cluster sample of region[l]	1238	45-74, men	(-) Education	--	Continuous
[Wener, 2000][*]<br>US national sample[a], cross-sectional, stratified multi-stage probability sample	22467	>4	--	(+) Lowest to highest: White, Hispanic, Black	Continuous
[Williams, 2004]<br>Dunedin, New Zealand, cross-sectional analysis within prospective birth cohort[n], births over 1 y period	822	26	Not presented	--	Continuous
			(n/s) SES, determined by occupation and income		
[Wong, 2001][*]<br>US national sample[a], cross-sectional, stratified multi-stage probability sample	9684	30-74	--	(+) Lowest to highest (men): White, Mexican-American, Black<br>(+) Lowest to highest (women): White, Black, Mexican-American	Continuous and categorical (<0.5, >=0.5-<1.0, >=1.0 mg/dL)

Table 1 legend:
(+) and (-) indicate statistically significant positive and negative associations, respectively, (n/s) indicates statistically non-significant associations
* Study reported on findings from unadjusted or minimally adjusted (for demographic variables) models only (n=11). [Wener, 2000] reported on individuals aged >4 and we report on participants aged >=20 y and without inflammatory conditions (N unknown).
** Results of unadjusted or minimally adjusted analyses appear on one row while those from studies including multivariable (fully adjusted) models appear on two rows (unadjusted or minimally adjusted analyses top, fully adjusted model bottom). Details for minimally adjusted models noted. Effect sizes discussed in Results section of text and in Table 2.
# Classification of race/ethnicity given as presented in original study
Continuous variables analyzed as *ln*CRP due to skewed distribution
[a] National Health and Nutrition Examination Survey (NHANES). Studies including the same years and age groups include partially overlapping samples. Studies used data from varying periods, as follows: NHANES 1988-1994: Abramson (2002), Danner (2003), Ford (2000 and 2002), Wener (2000), Wong (2001); NHANES 1999-2000: Ford (2003 and 2004); NHANES 1999-2002: Alley (2005).
[b] Hamilton, Toronto, Edmonton, the Six Nations Reservation (Oshweken, Ontario)
[c] Finnish Platelet Aggregation and Inflammation Study (PAIS)

[d] The Cardiovascular Risk in Young Finns Study
[e] Multi-Ethnic Study of Atherosclerosis (MESA): Baltimore, MD; Chicago, IL; Forsyth County, NC; Los Angeles County, CA; Northern Manhattan, NY; and St Paul, MN.
[f] British Women's Heart and Health Study: participants from 23 British towns
[g] Study of Women's Health Across the Nation (SWAN): Boston, MA; Chicago, IL; the Detroit area, MI; Newark, NJ; Pittsburgh, PA; Los Angeles and Oakland, CA
[h] The Chicago Health, Aging, and Social Relations Study
[i] The Caerphilly Prospective Heart Disease Study
[j] Turkish Adult Risk Factor Study. Study includes participants from three of seven regions included in cohort: Marmara, Aegean, and Mediterranean.
[k] The ATTICA Study. Overlapping sample populations (same individuals examined more than once).
[l] The KORA (Cooperative Health Research in the Region of Augsburg) Survey 2000, [Rathmann, 2006]; The MONICA (Monitoring of Trends and Determinants in Cardiovascular Disease) Augsburg Project, [Thorand, 2003]
[m] Midspan family study in Renfrew and Paisley, Scotland. [St J O'Reilly, 2006] also included data from West of Scotland coronary prevention study (WOSCOPS), which did not meet inclusion criteria and was excluded. Overlapping sample populations (same individuals examined more than once).
[n] Dunedin Multidisciplinary Health and Development Study
