# Supplementary material for: Socioeconomic and racial/ethnic differentials of C-reactive protein levels: a systematic review of population-based studies
Source: BMC Public Health. 2007 Aug 17;7:212. doi: 10.1186/1471-2458-7-212 (PMC2018719; doi:10.1186/1471-2458-7-212)
Supplement: Additional file 2 — Summary of results and effect sizes for the associations between socioeconomic factors and race/ethnicity with C-reactive protein (CRP) levels. The data provided summarize the effect sizes of the associations between C-reactive protein levels in adults in relation to socioeconomic factors or race/ethnicity from a systematic review of population-based studies. [file 1471-2458-7-212-S2.doc]

### Table 2: Summary of results and effect sizes for the associations between socioeconomic factors and race/ethnicity with C-reactive protein (CRP) levels.

| Author, year | Effect sizes for association with C-reactive protein | |
| --- | --- | --- |
|  | Socioeconomic factors | Race/ethnicity |
| Abramson, 2002 | Odds ratio^*^ of CRP> 0.66 mg/L:  <12 y of education= 1.66  12 y of education= 1.99  (>12 y education ref) | Odds ratio^*^ of CRP> 0.66 mg/L:  African-American= 1.75  “Other”= 0.58  (White ref) |
| Alley, 2005 | 50^th^ / 75^th^ / 90^th^ percentiles of CRP (mg/L):  In poverty= 2.3 / 5.6 / 13.4  Above poverty= 2.0 / 4.5 / 9.1 | Adjusted odds ratio^a^ (95% CI) for high CRP (3.1-10.0 mg/L):  Black= 1.33 (1.09, 1.62)  Mexican-American= 1.26 (1.01, 1.58)  Other= 1.10 (0.91, 1.32)  (White ref) |
|  | Fully adjusted odds ratio^a^ (95% CI) for CRP levels, those in poverty:  1.1-3.0 mg/L= 0.95 (0.80, 1.13)  3.1-10.0 mg/L = 0.88 (0.73, 1.06)  > 10 mg/L = 1.27 (1.01, 1.62)  (CRP ≤ 1.0 mg/L ref) | Fully adjusted odds ratio^a^ (95% CI) for high CRP (3.1-10.0 mg/L):  Black= 1.45 (1.16, 1.80)  Mexican-American= 1.19 (0.94, 1.53)  Other= 1.13 (0.93, 1.38)  (White ref) |
| Anand, 2004 | -- | Sex/age adjusted mean (SE) CRP levels (>10 excluded), mg/L:  Chinese= 1.18 (0.13)  European= 2.06 (0.12)  South Asian= 2.59 (0.12)  Aboriginal= 3.74 (0.14) |
|  |  | Fully adjusted^b^ mean (SE) CRP levels, mg/L:  Chinese= 1.72 (0.13)  European= 2.13 (0.12)  South Asian= 2.72 (0.12)  Aboriginal= 2.85 (0.15) |
| Bo, 2005 | Unadjusted effect size data not available | -- |
|  | Adjusted odds ratio^c^ (95% CI) for CRP≥ 3 mg/L according to years of education:  Secondary school= 0.85 (0.61, 1.19)  University= 0.99 (0.60, 1.64)  (Primary school ref) |  |

| Chambers, 2001 | -- | Age adjusted geometric mean (SD) CRP levels, mg/L:  European= 1.47 (1.62)  Indian Asian= 1.71 (1.81) |
| --- | --- | --- |
|  |  | Adjusted estimated percentage difference^d^ (95% CI) in CRP, Indian Asian-European= 4 (-10, 24) |
| Danner, 2003 | Prevalence ratio^*^ of CRP≥ 0.22 mg/dL among men / women:  < High school= 1.46 / 1.18  High school= 1.25 / 1.35  (At least some college ref) | Prevalence ratio^*^ of CRP≥ 0.22 mg/dL among men / women:  African-American= 1.57 / 1.44  Mexican-American= 1.24 / 1.44  “Other”= 1.34 / 1.12  (Non-Hispanic white ref) |
| Ford, 2000 | Mean years (SE) of education according to level of CRP:  ≤ 0.21 mg/L= 12.4 (0.1)  > 0.21-< 0.55 mg/dL= 12.0 (0.2)  ≥ 0.55 mg/dL= 11.3 (0.1) | -- |
| Ford, 2003 | Unadjusted effect size data not available | Age adjusted beta coefficient^e^ (SE) for *ln*CRP: African-American= 0.03 (0.09)  Mexican-American= 0.09 (0.10)  “Other”= 0.20 (0.25)  (White ref) |
|  | Fully adjusted beta coefficient^e^ (SE) for *ln*CRP:  < High school= 0.13 (0.10)  High school/equivalent= 0.07 (0.09)  (> High school ref) | Fully adjusted beta coefficient^e^ (SE) for *ln*CRP: African-American= -0.02 (0.09)  Mexican-American= 0.02 (0.11)  “Other”= 0.16 (0.21)  (White ref) |
| Ford, 2004 | Unadjusted effect size data not available | Median (IQR) of CRP levels:  White= 2.5 (0.9, 5.8)  African-American= 3.5 (1.1, 7.5)  Mexican-American= 3.6 (1.4, 7.7) |
|  | Adjusted beta coefficient^f^ (SE) for *ln*CRP:  < High school= 0.17 (0.09)  High school/equivalent= -0.04 (0.08)  (> High school ref) | Adjusted beta coefficient^f^ (SE) for *ln*CRP: African-American= -0.01 (0.09)  Mexican-American= 0.29 (0.07)  “Other”= 0.00 (0.13)  (White ref) |
| Forouhi, 2001 | Effect size data not available | Median (IQR) CRP (mg/L) of men / women:  European= 0.92 (0.34, 1.61) / 0.70 (0.41, 1.70)  South Asian= 1.07 (0.76, 1.50) / 1.35 (0.72, 3.04) |
| Jousilahti, 2003 | Age adjusted geometric mean of CRP (mg/L) according to socioeconomic status:  Low= 2.32  Middle= 1.90  High= 1.52 | -- |
|  | Fully adjusted^g^ geometric mean of CRP (mg/L) according to socioeconomic status:  Low= 2.11  Middle= 1.91  High= 1.63 |  |
| Kivimaki, 2005 | Age/sex adjusted mean (SE) CRP level (mg/L) according to socioeconomic indicators (lowest to highest level):  Parental occupation= 2.10 (0.13) / 1.91 (0.13) / 1.40 (0.20)  Own education= 1.97 (0.28) / 1.97 (0.10) / 1.52 (0.19) | -- |
|  | Fully adjusted beta coefficient^h^ with *ln*CRP for:  Parental occupation= -0.03 (0.03)  Own education= -0.04 (0.05) |  |
| Lakoski, 2006 | -- | Median (IQR) of CRP (mg/L) according to ethnicity in men / women:  Chinese= 0.80 (1.10) / 0.99 (1.50)  Caucasian= 1.30 (2.20) / 2.47 (4.10)  African-American= 1.80 (2.80) / 3.40 (6.10)  Hispanic= 1.93 (2.90) / 3.00 (5.00) |
|  |  | Adjusted geometric mean^i^ of CRP (mg/L) according to ethnicity in men / women:  Chinese= 0.95 / 1.20  Caucasian= 2.03 / 2.75  African-American= 2.12 / 3.19  Hispanic= 2.51 / 3.39 |
| Loucks, 2006 | Age/sex adjusted mean (95% CI) CRP (mg/L) according to education:  ≤ High school= 4.7 (4.5, 4.9)  Associate’s degree= 4.7 (4.5, 4.9)  Bachelor’s degree= 3.6 (3.4, 3.7)  >Bachelor’s degree= 3.2 (3.0, 3.3) | -- |
|  | Fully adjusted beta coefficient^j^ (95% CI) between years of education and *ln*CRP= -0.03 (-0.05, -0.02) |  |
| Matthews, 2005 | -- | Median (IQR) CRP (mg/L) according to ethnicity:  Japanese= 0.5 (0.2, 1.2)  Chinese= 0.7 (0.3, 1.4)  White= 1.4 (0.6, 3.9)  Hispanic= 2.3 (1.0, 5.1)  African-American= 3.0 (1.0, 7.2) |
|  |  | Adjusted^k^ effect size data not available |
| McDade, 2006 | Age/sex/ethnicity adjusted beta coefficient (SE) between education and *ln*CRP= -0.06 (0.03) | Median (IQR) CRP (mg/L) in men / women:  European-American= 0.59 (0.44, 1.50) / 1.05 (0.44, 1.88)  Latino-American= 1.00 (0.55, 1.65) / 1.49 (0.78, 3.10)  African-American= 1.07 (0.37, 1.70) / 3.30 (1.39, 4.47) |
|  | Fully adjusted^l^ beta coefficient (SE) between education and *ln*CRP= -0.05 (0.03) | Fully adjusted beta coefficient^l^ (SE) with *ln*CRP: African-American= 0.12 (0.09)  Latino-American= 0.08 (0.09)  (European-American ref) |
| Onat, 2001 | Partial coefficient correlation between *ln*CRP and family income:  Men= -0.06  Women= -0.10 | -- |
|  | Adjusted effect size data not available |  |
| Panagiotakos, 2004 | Mean (SD) CRP (mg/dL) according to education:  Low= 2.04 (1.04)  Medium= 1.42 (1.01)  High= 1.12 (1.71) | -- |
|  | Beta coefficient^m^ (SE) of education with *ln*CRP=  -0.012 (0.001) |  |
| Panagiotakos, 2005 | Mean (SD) CRP (mg/dL) according to tertile of socioeconomic index:  Low= 2.1 (1.0)  Medium= 1.5 (1.2)  High= 1.6 (1.7) | -- |
|  | Adjusted beta coefficient^m^ (SE) of socioeconomic index score with *ln*CRP= -0.10 |  |
| Rathmann, 2006 | Age-adjusted geometric mean (SD) CRP (mg/L) according to socioeconomic index in men / women:  Low= 1.66 (3.10) / 2.17 (2.70)  Intermediate= 1.75 (3.20) / 1.68 (2.60)  High= 1.49 (3.30) / 1.32 (2.60) | -- |
|  | Fully adjusted effect size data not available |  |
| Sattar, 2004 | Geometric mean CRP (mg/L) in men / women according to socioeconomic index:  Affluent= 0.70 / 0.70  Intermediate= 0.87 / 0.91  Deprived= 0.94 / 1.15 | -- |
| St J O'Reilly, 2006 | Age adjusted estimated percentage increase (95% CI) in CRP (mg/L) with one unit difference in deprivation category in two studies:  WOSCOPS= 8.3 (6.5, 10.2)  MIDSPAN men / women= 6.4 (1.4, 11.5) / 11.3 (6.2, 16.7) | -- |
|  | Fully adjusted^o^ estimated percentage increase (95% CI) in CRP (mg/L) with one unit difference in deprivation category in two studies:  WOSCOPS= 5.4 (3.6, 7.1)  MIDSPAN men / women= 2.3 (-2.2, 7.0) / 4.8 (0.4, 9.5) |  |
| Thorand, 2003 | Antilog mean (SE) CRP (mg/L) according to education:  <12 years= 1.53 (1.03)  ≥ 12 years= 12.6 (1.04) | -- |
| Wener, 2000 | -- | 95^th^ percentile (95% CI) of CRP (mg/dL) levels among men / women aged 20-39:  White= 0.65 (0.55, 0.75) / 1.16 (0.96, 1.36)  Hispanic= 0.69 (0.53,0.85) / 1.67 (1.23, 2.11)  Black= 0.94 (0.78, 1.10) / 1.65 (1.45, 1.85) |
| Williams, 2004 | Unadjusted effect size data not available | -- |
|  | Adjusted^p^ ratio change (95% CI) in CRP related to low socioeconomic status in men / women= 1.03 (0.90, 1.18) / 0.85 (0.70, 1.04) |  |
| Wong, 2001 | -- | Percent of men / women with CRP> 10 mg/L:  White= 5.5 / 10.3  Mexican-American= 5.1 / 14.2  Black= 9.1 / 16.3 |

Results of unadjusted or minimally adjusted analyses appear on one row while those from studies including multivariable (fully adjusted) models appear on two rows (unadjusted or minimally adjusted analyses top, fully adjusted model bottom). Results shown as given in original article unless otherwise noted.

Inadequate numerical data on effect size was not available from the following articles: Danesh (1999), Ford (2002), Khera (2005), Lawlor (2005)

^*^ Calculated from prevalence data appearing in article

^a^ Top row adjusted for age and poverty; full model (bottom row) adjusted for age, sex, poverty or race, recent illness, leukocyte count, asthma, chronic bronchitis, rheumatoid arthritis, obesity, smoking, heavy drinking, exercise (data from personal communication with the author)

^b^ Adjusted for age, sex, BMI, waist circumference, triglycerides, HDL, systolic BP, and HbA1c.

^c^ Adjusted for age, sex, BMI, smoking, alcohol intake, physical activity, living in rural area

^d^ Adjusted for age, smoking, diabetes, hypertension, hypercholesterolemia, social classs, waist to hip ratio, insulin resistance score

^e^ Adjusted for age, education, smoking status, body mass index, and alcohol intake

^f^ Adjusted for age, race, education, smoking status, total cholesterol concentration, systolic blood pressure, waist circumference, alcohol use, hormone replacement therapy

^g^ Adjusted for age, smoking, waist to hip ratio, prevalent longstanding diseases

^h^ Adjusted for age, sex, smoking, physical activity, BMI, waist to hip ratio, systolic and diastolic blood pressure, insulin, glucose, HOMA, HDL and LDL cholesterol, triglycerides

^i^ Adjusted for age, BMI, diabetes, hypertension, smoking, alcohol use, HMG-CoA reductase inhibitors, aspirin use, estrogen medications, physical activity, LDL and HDL cholesterol

^j^ Adjusted for age, sex, smoking, systolic and diastolic blood pressure, total cholesterol:high density lipoprotein cholesterol ratio, BMI, lipid-lowering medication, antihypertensive medication, prevalent cardiovascular disease, depression

^k^ Adjusted for study site, education, leisure physical activities, total calories, percent calories from fat

^l^ Adjusted for age, sex, ethnicity or education, waist circumference, time to fall asleep

^m^ Adjusted for age, sex, smoking, compliance to medication, BMI, diet score, physical activity

^n^ Adjusted for age, smoking, BMI, waist circumference, alcohol intake, physical activity

^o^ Adjusted for age, smoking, BMI, medication

^p^ Adjusting for systolic blood pressure, apolipoproteins A1 and B, health problems and oral contraceptive use
